# Supplementary material for: The causal role of circulating inflammatory markers in osteoporosis: a bidirectional Mendelian randomized study
Source: Front Immunol. 2024 Jul 18;15:1412298. doi: 10.3389/fimmu.2024.1412298 (PMC11291241; doi:10.3389/fimmu.2024.1412298)
Supplement: Supplementary file 1 [file DataSheet_1.zip › supplementary material/OSTEOPOROSIS-CIM/GCST90274788.tsv.gz_leave_one_out.pdf]

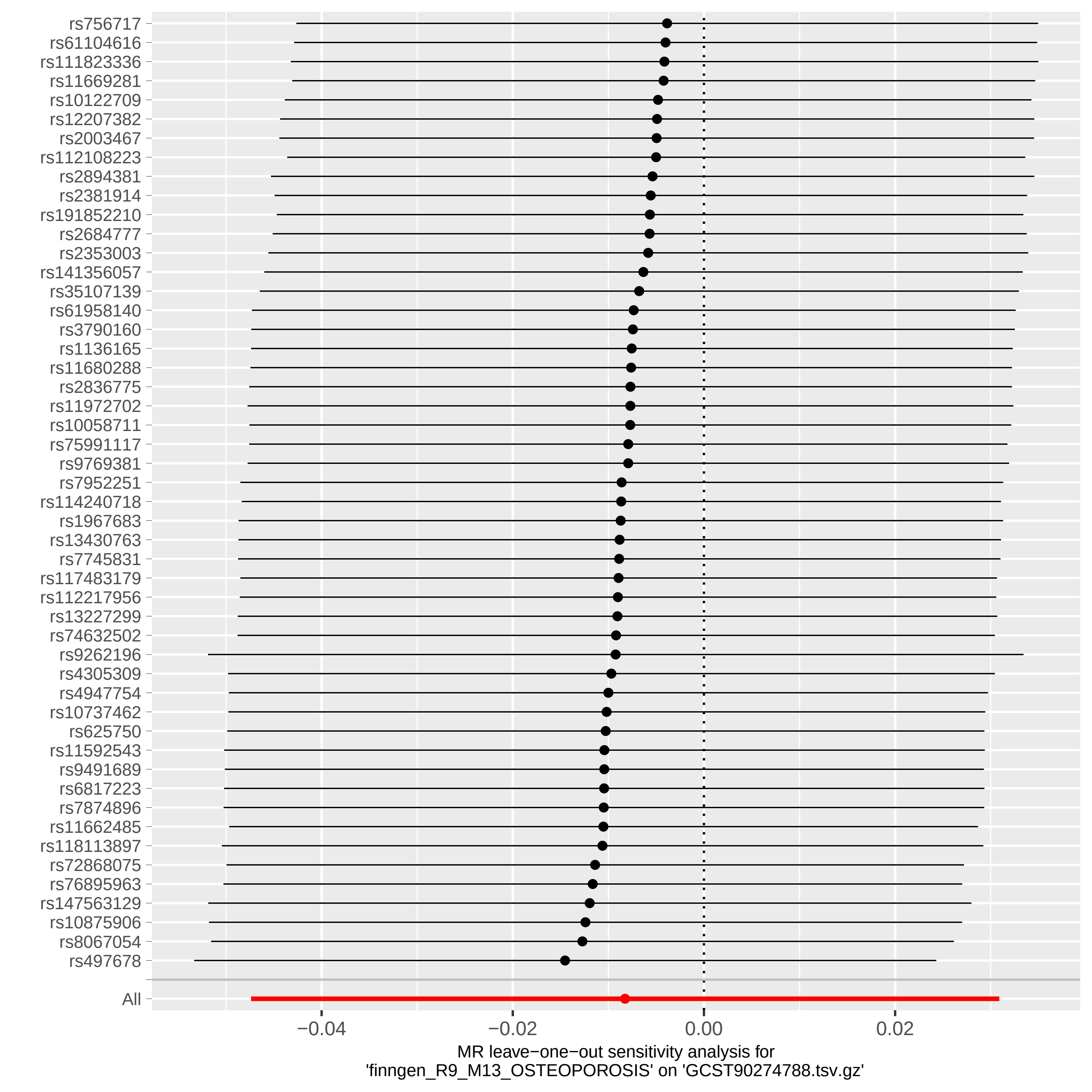

rs756717
rs61104616
rs111823336
rs11669281
rs10122709
rs12207382
rs2003467
rs112108223
rs2894381
rs2381914
rs191852210
rs2684777
rs2353003
rs141356057
rs35107139
rs61958140
rs3790160
rs1136165
rs11680288
rs2836775
rs11972702
rs10058711
rs75991117
rs9769381
rs7952251
rs114240718
rs1967683
rs13430763
rs7745831
rs117483179
rs112217956
rs13227299
rs74632502
rs9262196
rs4305309
rs4947754
rs10737462
rs625750
rs11592543
rs9491689
rs6817223
rs7874896
rs11662485
rs118113897
rs72868075
rs76895963
rs147563129
rs10875906
rs8067054
rs497678
All
−0.04
−0.02
0.00
0.02
MR leave−one−out sensitivity analysis for
'finngen_R9_M13_OSTEOPOROSIS' on 'GCST90274788.tsv.gz'
